# Pennsylvania

**Published:** 1896-04

**Authors:** 


### PENNSYLVANIA.

THE State Board of Veterinary Medical Examiners beg leave to make the following statements pertinent to the granting of license and for registration to practice veterinary medicine in this State in accordance with the Act creating this Board:

1. All practitioners legally registered and qualified in any county of this State in accordance with the Registration Act of 1889 are not amenable to this law, providing such registration was made prior to the first Monday in September, 1895.

2. Such registration in any one county is sufficient for the entire State.

3. Any person who wishes to enter upon the practice of veterinary medicine and surgery after the first Monday in September, 1895, cannot register as a qualified practitioner without passing an examination by and receiving a license from the Board, as prescribed by the Act.

4. After the first Monday in September, 1895, no prothonotary is vested with authority to register any applicant unless he be provided with a license from the Examining Board. All registrations must be made through the Board.

5. Persons from other States wishing to practice in this Commonwealth are not qualified without receiving a license from the Veterinary Board according to Section 8 of the Act.

6. Applicants receiving their degree in veterinary medicine after July 1, 1896, must be graduates of a school with a three-years' curriculum of at least six months each.

SIMON J. J. HARGER, President. W. HORACE HOSKINS, Secretary.

PHILADELPHIA, December 20, 1895.

The regular meeting of the State Board of Veterinary Medical Examiners will be held at Philadelphia on the third Monday and the day following of this month. All information may be obtained by applying to the Secretary, W. Horace Hoskins, 3452 Ludlow Street, Philadelphia.

Allegheny City's council has just passed an ordinance establishing the position of city veterinarian at a salary of $1000 per year. The mayor of Allegheny has been petitioned to remove the present head of the Board of Health, who is a layman, and appoint a competent physician for the place. This movement promises to be successful, and a department of bacteriology and other radical reform measures are under consideration.

